# The role of habitat choice in micro‐evolutionary dynamics: An experimental study on the Mediterranean killifish *Aphanius fasciatus* (Cyprinodontidae)

**DOI:** 10.1002/ece3.3540

**Published:** 2017-11-01

**Authors:** Dario Angeletti, Claudia Sebbio, Alessandro Carlini, Claudia Strinati, Giuseppe Nascetti, Claudio Carere, Roberta Cimmaruta

**Affiliations:** ^1^ Department of Ecological and Biological Sciences Ichthyogenic Experimental Marine Center (CISMAR) Tuscia University Tarquinia VT Italy; ^2^ Department of Monitoring of Environmental Quality Italian National Institute for Environmental Protection and Research (ISPRA) Rome Italy

**Keywords:** behavioral genetics, environmental heterogeneity, evolution, evolutionary ecology, genetic divergence, genetic structure, population genetics

## Abstract

Habitat choice is defined as a nonrandom distribution of genotypes in different microhabitats. Therefore, it could exert a great impact on the genetic variance of natural populations by promoting genetic divergence, local adaptation, and may even lead to sympatric speciation. Despite this potential role in micro‐ and macro‐evolutionary processes, there is little empirical evidence that the various genotypes within a population may differ in habitat choice‐related behaviors. Here, we tested whether habitat choice may have contributed to genetic divergence within a local population of the Mediterranean killifish *Aphanius fasciatus*, which emerged between groups inhabiting microhabitats with different oxygen concentrations during previous field studies. In a first experiment, we studied the distribution of individuals in conditions of hypoxia and normoxia to test whether they had a different ability to shy away from a hypoxic environment; in a second experiment, we analyzed the individual behavior of fish separately in the two conditions, to verify whether they showed peculiar behavioral responses linked to a possible differential distribution. We then analyzed the six allozyme loci, whose allelic and genotypic frequencies were significantly divergent in the previous studies. In the first test, we found that the distribution of the two homozygote genotypes of the glucose‐6‐phosphate isomerase‐1 locus (GPI‐1) was significantly different between the hypoxic and the normoxic conditions. During the second test, all individuals were more active in hypoxic conditions, but the two GPI‐1 homozygotes showed a significant difference in time spent performing surface breathing, which was consistent with their distribution observed in the first experiment. These results provide evidence that individual behavioral traits, related to genetic features, may lead to a nonrandom distribution of genotypes in heterogeneous although contiguous microhabitats and, consequently, that habitat choice can play a significant role in driving the micro‐evolutionary dynamics of this species.

## 1 INTRODUCTION

The patterns of genetic variability displayed by natural populations are typically considered to result from the action of evolutionary forces. As mutational events create new alleles in a local population, allelic and genotypic frequencies may change randomly, via genetic drift, or in an adaptive way by natural selection (Edelaar & Bolnick, 2012). Divergence and local genetic adaptation can occur when drift or selection overcome the homogenizing effect of gene flow, which is assumed to be high, thus preventing differentiation at small spatial scale (Hendry, Day, & Taylor, 2001; Björklund, Aho, & Larsson, 2007; Adams et al., 2016). Concurrently, gene flow may extend the new genotypic traits acquired in a local population to the adjacent ones, which also contrasts divergence in this case (Edelaar & Bolnick, 2012). Thus, gene flow is generally considered a homogenizing force, because theory often assumes that migrants carry a random sample of regional alleles (Lenormand, 2002). Nevertheless, if genotypes differ in dispersal ability or in habitat preference, this may lead them to a nonrandom distribution, and local differentiation can be facilitated rather than impeded by gene flow (Bolnick & Otto, 2013). This nonrandom distribution of genotypes in different microhabitats is known as habitat choice and, in addition to evolutionary forces, may have a great influence in evolutionary processes (Edelaar, Siepielski, & Clobert, 2008; Canestrelli, Bisconti, & Carere, 2016).

Habitat choice can be operationally defined as any behavior that leads some individuals to spend more time in one habitat type than in another, with respect to the expectation of a random allocation (Futuyma, 2001; Webster, Galindo, Graham, & Butlin, 2012). If those individuals displaying a different behavior and distribution differ for some genotypic features with respect to the others, this can give rise to a genetic divergence among groups occupying different and contiguous habitats. Moreover, if the spatial segregation of genotypes involves matching phenotypes to habitats conferring higher fitness, genetic polymorphism can be maintained in heterogeneous habitats, and microgeographic adaptation can result (Hedrick, 1986; Ravigné, Dieckmann, & Olivieri, 2009; Bolnick & Otto, 2013; Richardson, Urban, Bolnick, & Skelly, 2014). Over time, genetic divergence and local adaptation can favor assortative mating within local groups, which reinforces the divergence itself and favors the arising of reproductive isolation barriers (Servedio & Noor, 2003). Thus, habitat choice could not only explain part of the genetic polymorphism displayed by the natural populations but it offers some of the greatest potential for promoting microgeographic adaptation (Richardson et al., 2014), and it can also initiate processes of sympatric speciation (Maynard Smith, 1966).

Despite its potential role in micro‐ and macro‐evolutionary processes and the increasing recognition that genotypes can differ in dispersal ability or in habitat preference (Rice, 1984; Canestrelli et al., 2016; Forsman & Berggren, 2017), habitat choice is still an overlooked issue in evolutionary ecology (Edelaar et al., 2008). Indeed, habitat choice has been rarely evoked to explain the genetic patterns of natural populations (e.g., Byers, 1980, 1983; Borowsky, 1990; Szarowska, Falniowski, Mazan, & Fialkowski, 1998), likely because field studies make it difficult to disentangle the patterns of genetic divergence originated by habitat choice from those due to the action of other diverging forces, such as drift or selection. Convincing empirical evidence has been obtained in *Drosophila* and other invertebrates (Jones & Probert, 1980; Jones, 1982; Hoffmann, Parsons, & Nielsen, 1984; Barker, Vacek, East, & Starmer, 1986; De Meeûs, Hochberg, & Renaud, 1995), but so far, experimental evidences of genetically related habitat choice remain scarce, especially in vertebrates.

**Figure 1:**
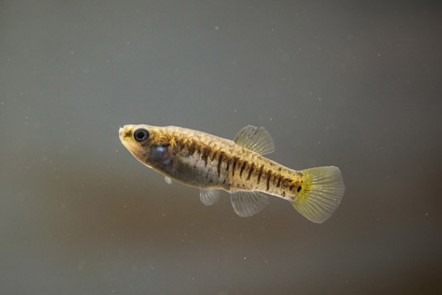
Specimen of *Aphanius fasciatus* collected in the Tarquinia saltworks (see Sections 1 and 2 for further details on species and sampling)

As spatial segregation is only feasible within the typical dispersal range of individuals that must sample alternate habitats (Richardson et al., 2014), a good organism to study habitat choice should occupy strongly environmentally heterogeneous habitats and have a mobility comparable to the extension of the spatial heterogeneity. Moreover, unstable environments that reach limit conditions in some patches can force individuals to migrate (Hoffmann & Hercus, 2000), likely exalting possible differences in their dispersal ability. The Mediterranean killifish *Aphanius fasciatus* (Figure 1) has these features: It is a highly gregarious and euriecial species, which lives in heterogeneous and unstable environments, such as coastal brackish waters, lagoons, and salt marshes, but is also a mobile species within its habitat, even if local populations are strongly isolated because of habitat discontinuity (Sebbio et al., 2014; Pappalardo, Gonzalez, Tigano, Doadrio, & Ferrito, 2015; Cavraro, Torricelli, & Malavasi, 2013; Cavraro et al., 2017).

In previous studies, we demonstrated that the extreme environmental conditions reached during a period of eutrophication, generated a genetic divergence in the allelic and genotypic frequencies at six allozyme loci at a microgeographic scale, in the local population of the killifish inhabiting the Tarquinia saltworks (central Italy). Our analysis indicated that part of the genetic divergence, which emerged in the face of gene flow, was the result of the combined action of selection and drift, promoted by high salinity and hypoxia (Angeletti, Cimmaruta, & Nascetti, 2009, 2010; Angeletti et al., 2017; Cimmaruta, Angeletti, Pontremolesi, & Nascetti, 2010). In particular, we detected different signatures at different allozyme markers, providing evidence for either selection (adenosine deaminase, ADA), and drift (glucose‐6‐phosphate isomerase‐3, GPI‐3; phosphoglucomutase‐1, PGM‐1). For other loci (mannose‐6‐phosphate isomerase, MPI; glucose‐6‐phosphate isomerase‐1, GPI‐1; phosphoglucomutase‐2, PGM‐2), the possible dynamics generating the divergence remained unidentified, and we hypothesized that habitat choice might have played a role in promoting or copromoting it, as divergence emerged even between groups sampled very closely to each other in microhabitats with different oxygen concentrations (see Angeletti et al., 2009). Although the mechanisms are unclear, fish seem able to detect, cope with behavior, and avoid hypoxia (Pihl, Baden, & Diaz, 1991; Wu, 2002). A typical behavioral change observed in species that regularly experience hypoxic conditions is an increase in both activity and surface breathing, (i.e., the individual swims close to the surface in upright posture where it increases the ventilation rate of the gills with the relatively oxygenated water in contact with the air; Petersen & Petersen, 1990; Verheyen, Blust, & Decleir, 1994; Killen, Marras, Ryan, Domenici, & McKenzie, 2012). We therefore predicted that variation in the expression of these behaviors could be related to a differential individual ability of certain genotypes in actively coping with a hypoxic environment. In other words, we hypothesized that there might be a relationship between the behaviors displayed by fishes when facing hypoxia and the genotype at one or more allozyme markers presenting divergence in the field, which led to a nonrandom distribution of genotypes between contiguous conditions of hypoxia and normoxia.

To verify this hypothesis, we performed behavioral experiments in controlled conditions of hypoxia and normoxia, and we then analyzed the individual genotypic traits of the animals for all six aforementioned allozyme loci. With this combined behavioral and genetic approach, we aimed to test: (1) the differences in fish behavior between normoxic and hypoxic conditions; (2) whether individuals and genotypes showed differences in their ability to shy away from a hypoxic environment by assessing any differential distribution in conditions of hypoxia and normoxia; (3) whether the variation in the behavioral response to hypoxia (activity levels, surface breathing, and posture) was related to the different genotypes and could be implicated in their differential distribution.

## 2 MATERIALS AND METHODS

### 2.1 Sampling

Protocol and experimental procedures were approved by the University of Tuscia ethical committee and conducted within the EU legislation for the protection of animals used for scientific purposes (Directive 2010/63/EU). We performed three samplings in the Tarquinia saltworks (Cimmaruta, Blasi, Angeletti, & Nascetti, 2010 and Bellisario et al., 2010, 2013 for details on the sampling area). Samplings were performed in autumn (28 November 2013) and spring (27 March 2014 and 12 May 2014, respectively), using fish traps and collecting 131, 123, and 121 individuals of *Aphanius fasciatus*, respectively (total *n* = 375). Immediately after capture, the individuals were transported alive to the laboratory, using plastic bags filled with water from the sampling point. The fish from each sampling group were then placed in the first experimental aquarium (described below) and left there overnight for at least 12 hr of acclimation. After acclimation, we tested each sampling group in separate experimental sessions, performing one after the other for the two following experiments.

### 2.2 First experiment: distribution of individuals and genotypes

To test the ability of genotypes (i.e., individuals) to shy away from a hypoxic environment and how they distributed within conditions of hypoxia and normoxia, we performed the first experiment on each of the three groups of specimens collected. We prepared an aquarium (120L × 40W × 50H cm) separated into two compartments by a glass in the middle. At the center of the glass, there was a hole with a screw cap (5 cm in diameter) to open/close the connection between the two parts of the tank. The water conditions in the two compartments were maintained as follows: 42 ± 1 ‰ salinity, 23 ± 1°C water temperature (using two thermostats for the aquarium), and 5–6 mg/L (80% of saturation) oxygen concentration. The parameters were monitored using a refractometer and field probes (Oxyguard, Denmark). During acclimation, fish were placed in a compartment, while the hole was kept open to enable fish to freely explore the habitat. After acclimation, fish were gently pushed in one compartment and the hole was closed. In the compartment containing the fish, the oxygen concentration was progressively lowered up to 0.5–1 mg/L (between 10% and 15% of saturation), by delivering nitrogen gas for food use in the water (Aligal^™^ 1, Air Liquide, Italy), through a porous stone for aquariums. Then, the hole was opened again, and the fish were left to freely distribute between the two compartments for 4 hr before closing the hole, so that the fish were in the compartment of their “choice” at the end of the experiment. The number of individuals allocated in the experimental aquarium minimizing the stress and the cut‐off time of 4 hrs was set based off of previous pilot experiments: No deaths occurred and no aggressive or abnormal reactions were observed at the densities used for the experiments; moreover, we observed that after the first three 3 hr of free distribution, the passage of fish between the two compartments became rare. During the experiment, similar flows of nitrogen and air were delivered to the two compartments to constantly maintain the difference in oxygen concentrations and ensure similar levels of noise in the two compartments. Any other visual or noise disturbance was avoided during the experiments by curtains placed around the aquarium. At the end of the experiments, the delivery of nitrogen and air in the two compartments was stopped, and the aquarium was opened on the top, to allow oxygen to reach a similar concentration in the two compartments (5–6 mg/L 80% of saturation), before starting the second experiment after about 15 min.

### 2.3 Second experiment: individual behavioral tests

After the end of the first experiment with each of the three sampling groups, we started to test a subsample of the group (31 from the first group, 43 from the second, and 55 from the third; total: 129), to study the behavioral traits of fish that could justify their distribution in the previous test. We prepared an aquarium (60L × 40W × 50H cm) separated into two compartments by a glass in the middle. In the two compartments, the same conditions of the previous experiment were replicated, that is, 42 ± 1‰ salinity and 23 ± 1°C water temperature in both compartments, while the oxygen concentration was maintained at 5–6 mg/L in the normoxic compartment and at 0.5–1 mg/L in the hypoxic one. Of the 129 fish tested in this second experiment, 64 had been found in the normoxic compartment at the end of the first experiment and 65 in the hypoxic one. Each fish was individually tested for 13 min in the normoxic compartment and, immediately after, for 13 min in the hypoxic one, while avoiding any visual or noise disturbances during the tests. The individual tests were performed in the following 3–4 days in the same conditions of room light and temperature. The sequence of the two tests was inverted for 64 (32 from the normoxic compartment of the first experiment and 32 from the hypoxic one) of the 129 individuals and we conducted the tests alternating individuals coming from the two compartments of the first experiment. We captured and transferred each fish using an aquarium net and, after allowing a brief acclimation of 5 min after each transfer, the fish were filmed by a fixed camera placed in front of the aquarium, and the behavior of each fish was subsequently analyzed using the software OBSERVER 2.0 (Noldus, 1991). We considered the following parameters expected to be affected by change in oxygen levels: time spent in *activity*, defined as locomotion in any direction; time spent in *surface breathing*, defined as breathing activity (visually detected by clear regular movements of the branchial operculum) occurring at the surface of the water; time spent in *normal posture*, when the head and the caudal fin are in the same horizontal line; time spent in *upright posture*, when the head is higher than the caudal fin.

### 2.4 Morphometric and genetic data

Immediately after the two experiments, each individual was euthanized by immersion in an anesthetic solution (10 mg/L MS‐222 for light anesthesia followed by 500 mg/L MS‐222 for euthanasia). We then proceeded to sex assignment (based on the sexual dimorphism of the species; Leonardos & Sinis, 1999) and labeled each specimen with a unique identification number. We measured total body length (the straight‐line distance from the tip of the snout to the tip of the caudal fin), body height (the maximum straight‐line distance from the ventral margin to the dorsal one measured in front of the dorsal fin; Barnabe, 2003), and the weight of each fish, using a calliper (±0.01 mm of accuracy) and a digital scale (±0.01 g of accuracy). We then calculated a body condition index by applying a principal component analysis (PCA) to length, height, and weight (as e.g., in Fusani, Cardinale, Carere, & Goymann, 2009 and Kocovsky, Sullivan, Knight, & Stepien, 2013), which was then used for further analyses (Section 3). PCA is a multivariate statistical approach that reduces the dimensionality of the dataset by replacing multiple inter‐related original variables with a few, new uncorrelated component variables called “factors.” In our case, it served to reduce multiple testing to a single variable, instead of three (for a detailed description see Giuliani et al., 1994; Giuliani, Zbilut, Conti, Manetti, & Miccheli, 2004; Carere et al., 2015; Mojekwu & Anumudu, 2015). The morphometric characteristics of the three sampling groups are shown in Table 1, together with those of the three groups as pooled; PCA results are shown in Table S1. Fish were then frozen at −80°C until the allozyme analysis. Standard horizontal starch gel electrophoresis was performed at 5°C at 7–9 V/cm for 4–7 hr using muscle tissue, according to the methods described for this species by Cimmaruta, Scialanca, Luccioli, and Nascetti (2003). Six putative gene loci were analyzed: adenosine deaminase (ADA, E.C. 3.5.4.4); mannose‐6‐phosphate isomerase (MPI, E.C. 5.3.1.8); glucose‐6‐phosphate isomerase (GPI‐1 and GPI‐3, E.C. 5.3.1.9); and phosphoglucomutase (PGM‐1 and PGM‐2, E.C. 5.4.2.2). Isozymes were numbered in order of decreasing mobility from the most anodal, while allozymes were named numerically according to their mobility relative to the most common allele (=100). Thus, at the end of the two experiments, we obtained a dataset composed of the following variables for each individual: group of provenience (one of the three samples), sex, condition index, distribution in hypoxic or normoxic conditions (first experiment), measures of activity, surface breathing and posture (for the subsample of 129 individuals tested in the second experiment), and the genotype at the six allozyme loci.

**Table 1:** Morphometric data of the three samples of *Aphanius fasciatus* collected in the Tarquinia saltworks (Groups 1–3) and those of the three samples pooled (all)

Sampling group	N	NF/NM	ML (cm) ± *SE*	MH (cm) ± *SE*	MW (g) ± *SE*	MCI ± *SE*
Group 1	131	99/32	3.96 ± 0.04	0.75 ± 0.01	0.94 ± 0.03	−0.17 ± 0.09
Group 2	123	80/43	3.97 ± 0.05	0.75 ± 0.01	0.99 ± 0.04	−0.10 ± 0.10
Group 3	121	78/43	4.05 ± 0.07	0.79 ± 0.02	1.15 ± 0.07	0.20 ± 0.16
All	375	257/118	3.99 ± 0.03	0.76 ± 0.01	1.03 ± 0.03	−0.03 ± 0.07

*N*, number of specimens; NF/NM, number of females/number of males; ML, mean body length; MH, mean body height; MW, mean weight; MCI, mean body condition index; *SE*, standard error.

### 2.5 Data analysis

#### 2.5.1 Preliminary analysis

Before pooling the data from the three groups tested in the first experiment, we compared the sex ratio, the condition index, the allelic and the genotypic frequencies of the three groups to each other. For the same purpose, we repeated these analyses among the three groups of fish found in the normoxic compartment and among the three groups found in the hypoxic one at the end of the experiments. We performed a similar analysis to test whether the subsample of the second experiment (129 individuals) was representative of the entire sample (375 individuals) comparing the sex ratio, the condition index, and the allelic and genotypic frequencies of the subsample versus those of the entire sample. The check was performed applying a chi‐square test for the sex ratio, a *t* test for the condition index, and using exact probability tests for sample pairs as implemented in GENEPOP 3.4 (Raymond & Rousset, 1995; http://www.cefe.cnrs-mop.fr) for allelic and genotypic frequencies (see below for further details). The significance threshold was set at α* *= .0167, after Bonferroni correction for multiple tests (three tests). As no significant differences emerged from the aforementioned analyses (.11 ≤ *p *≤* *.99), we pooled the data of the three groups tested in the first experiment, and we considered the 129 individuals tested in the second experiment as being representative of the entire sample. We finally tested for genotypic linkage disequilibrium using Fisher's exact test implemented in GENEPOP 3.4, and no evidences emerged (*p *≥* *.29); thus, the genotypes at each locus could be considered independent from the genotypes at other loci.

#### 2.5.2 First experiment

After the experiment, we first calculated the proportion of individuals that moved to the normoxic compartment. We then checked for possible differences in the sex ratio and in the condition index among groups found in the two compartments after distribution by applying a chi‐square test and a t test, respectively. Subsequently, we obtained the allelic and genotype frequencies of the entire sample and of the two groups after the distribution using the BIOSYS‐2 software (Swofford & Selander, 1999). To test for allelic and genotypic differentiation between the two groups of individuals found in the two compartments, the distributions of allelic and genotypic frequencies were checked using exact probability tests for pairs of samples, as implemented in GENEPOP 3.4. An unbiased estimate of the *p* value for Fisher's exact test was performed for each locus and over all loci. We finally performed a multinomial logistic regression (MLR) to check for possible effects of the source group (one of the three samples), sex, condition index, and genotype at the six loci on the distribution in the two compartments. The MLR analysis was performed using the software SPSS (IBM Statistics, Italy), with a significance threshold at α* *= .05.

#### 2.5.3 Second experiment

We performed a t test for dependent samples (behavioral data were normally distributed) to test for behavioral differences between the normoxic and the hypoxic conditions, considering either each subsample (31 from the first group, 43 from the second, and 55 from the third) and the three subsamples pooled (129 individuals). We then tested for possible correlations among behavioral parameters within each of the two conditions by applying the Pearson's correlation coefficient, with the exclusion of the correlation between normal and upright posture, as they were mutually exclusive behaviors. Moreover, we performed a factorial ANOVA to check for the influence of sex, condition index, distribution in the first experiment, and genotype of each single locus on the behavioral parameters recorded in each of the two conditions. To further test whether sex and condition index have any influence on fish behavior, we compared the behavior of males with that of females (intersexual comparisons), and we tested for possible correlations among behavior and condition index in both hypoxia and normoxia. These latter tests were performed applying a t test and a Pearson's correlation coefficient, respectively. The tests were performed using the software SPSS (IBM Statistics, Italy), with a significance threshold at α* *= .05, except for the correlations among behavioral parameters and between behavioral parameters and the condition index (Pearson's tests) where we applied the Bonferroni correction, setting the significance thresholds at α* *= .01 (five tests) and α* *= .0125 (four tests), respectively.

## 3 RESULTS

### 3.1 First experiment: distribution of individuals and genotypes

**Figure 2:**
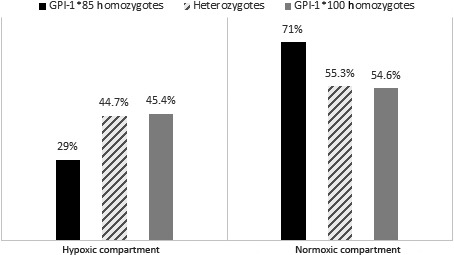
Percentages of the three GPI‐1 genotypes in the hypoxic and in the normoxic compartments, after the distribution of individuals

At the end of first experiment, after 4 hr of connection between the two compartments, 219 of the 375 individuals (58.4%) had moved to the normoxic compartment. The original sex ratio, which was similar to those reported in the literature for this species (Leonardos & Sinis, 1998), was maintained after the distribution of individuals: We counted 257 females (68.5%) versus 118 males (31.5%) in the entire sample, while after the free compartment choice, 154 females (70.3%) versus 65 males (29.7%) were in the normoxic compartment, and 103 females (66%) versus 53 males (34%) were in the hypoxic one. No significant differences emerged between the two groups after distribution (χ^2^ = 0.78; *p *=* *.38). The mean condition index was 0.11 in the normoxic compartment and −0.16 in the hypoxic one without any significant difference among them (*t* test: *p *=* *.08). Table 2 shows the allele frequencies recorded in the entire sample and in the two compartments after the distribution of individuals. In agreement with the previous studies (Angeletti et al., 2010, 2017), the six loci showed between two to three alleles. The GPI‐1 locus showed significant differences in the allelic and genotypic frequencies between the two compartments (*p *=* *.036 and *p *=* *.048, respectively), with a higher frequency of the GPI‐1*85 allele in the normoxic one, whereas no significant differences emerged for the other loci and for all loci (*p *≥* *.56). The differences at this locus were mostly due to the GPI‐1*85 homozygotes, as 49 out of 69 (71.0%) passed from the hypoxic compartment to the normoxic one, against 99 of the 176 heterozygotes (55.3%), and 71 of the 130 GPI‐1*100 homozygotes (54.6%; Figure 2). Consistently, a significant effect of the GPI‐1 locus on distribution emerged from the MLR analysis (Log = 760.15; χ^2^ = 5.77; *p *=* *.05), which was significantly higher for the GPI‐1*85 homozygotes with respect to both heterozygotes (χ^2^ = 4.52; *p *=* *.03) and GPI‐1*100 homozygotes (χ^2^ = 5.06; *p *=* *.02), confirming that a significantly higher proportion of GPI‐1*85 homozygotes migrated into the normoxic compartment. The MLR analysis did not yield any other significant result. In particular, the distribution of individuals was not influenced by the group of provenience, sex, condition index, or genotype at the other loci (*p *≥* *.23).

**Table 2:** Allele frequencies at the six polymorphic loci recorded in the entire sample (ES) and for the two groups of *Aphanius fasciatus* after the distribution of individuals under the hypoxic (HYX) and the normoxic (NOX) compartments

Locus	Allele	Allele frequencies		
		ES (*n* = 375)	HYX (*n* = 156)	NOX (*n* = 219)
ADA	100	0.714	0.702	0.723
	108	0.286	0.298	0.277
MPI	100	0.552	0.564	0.544
	104	0.448	0.436	0.456
GPI‐1	85	0.419	**0.375**	**0.449**
	100	0.581	**0.625**	**0.550**
GPI‐3	100	0.947	0.942	0.950
	108	0.053	0.058	0.050
PGM‐1	90	0.177	0.175	0.179
	94	0.348	0.344	0.351
	100	0.475	0.481	0.470
PGM‐2	95	0.014	0.010	0.010
	100	0.986	0.990	0.990

The only significant comparison was at GPI‐1 between HYX and NOX (in bold).

### 3.2 Second experiment: individual behavioral tests

**Figure 3:**
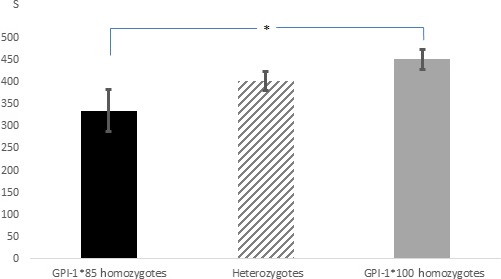
Mean time spent in surface breathing by the three GPI‐1 genotypes in hypoxia. A significant difference emerged between the two homozygous genotypes

**Figure 4:**
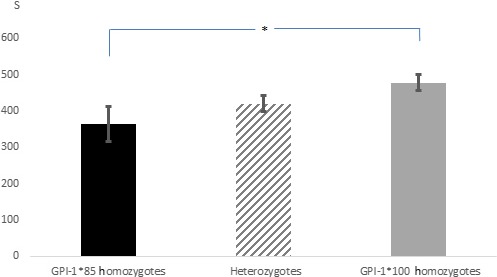
Mean time spent in upright posture by the three GPI‐1 genotypes in hypoxia. A significant difference emerged between the two homozygous genotypes

The t test for dependent samples showed significant differences among the duration of all the behavioral parameters registered under the two conditions with the exception of activity for the subsample of the second group tested (Table 3). Considering the results concerning all 129 specimens tested, the individuals spent more time in activity, surface breathing, and upright posture under hypoxia (*p *<* *.0001). As expected, in both hypoxia and normoxia, surface breathing positively correlated with upright posture (*r* = .75; *p *<* *.0001 and *r* = .48; *p *=* *.0005, respectively) and inversely correlated with normal posture (*r* = −0.75; *p *<* *.0001 and *r* = −.25; *p *=* *.004, respectively). Under normoxia, activity correlated with normal posture (*r* = .35; *p *<* *.0001) and it was inversely correlated with upright posture (*r* = −.33; *p *=* *.0001). No other correlation emerged from the Pearson's test (*p *≥* *.11). The factorial ANOVA did not show any effect of sex, condition index, and distribution in the previous experiment on individuals’ behavior, in either of the two conditions (*p *≥* *.32). Consistently, no difference between the behavior of males and females and no correlations among behavior and condition index emerged in any of the two experimental conditions (*t* test: *p *≥* *.21; Pearson's test: *p *≥* *.48, respectively). Conversely, the factorial ANOVA pointed out that the genotype at the GPI‐1 locus had a significant effect on surface breathing (*F *=* *3.535; *p *=* *.03) and upright posture (*F *=* *3.272; *p *=* *.04) in hypoxia; the post hoc analysis showed that under hypoxia, the GPI‐1*100 homozygotes spent significantly more time in surface breathing (*p *=* *.018; Figure 3) and in upright posture (*p *=* *.026; Figure 4) compared to GPI‐1*85 homozygotes. The other loci did not show any significant association with individuals’ behavior in the two conditions, and no interactions emerged (*p *≥* *.11).

**Table 3:** Measures of the behavioral parameters registered for the three subsamples of *Aphanius fasciatus* (Groups 1–3) and for the three subsamples pooled (All)

Subsample	Behavioral parameter	Mean time (s) under hypoxia ± *SE*	Mean time (s) under normoxia ± *SE*	*t* test		
				*t* value	*df*	*p*‐value
Group 1 (*N* = 31)	Surface breathing	383.69 ± 37.03	11.12 ± 9.39	9.809	30	**<.0001**
	Activity	315.92 ± 32.55	189.39 ± 31.15	3.141	30	**.0037**
	Upright posture	484.57 ± 31.47	105.09 ± 27.03	9.664	30	**<.0001**
	Normal posture	212.83 ± 28.78	601.66 ± 27.19	−10.508	30	**<.0001**
Group 2 (*N* = 43)	Surface breathing	389.78 ± 30.36	3.65 ± 1.97	12.826	42	**<.0001**
	Activity	441.11 ± 24.71	412.13 ± 33.64	0.817	42	.418
	Upright posture	387.12 ± 33.37	30.93 ± 14.14	10.279	42	**<.0001**
	Normal posture	316.25 ± 30.79	689.02 ± 14.14	−11.466	42	**<.0001**
Group 3 (*N* = 55)	Surface breathing	442.98 ± 17.36	9.94 ± 6.75	24.672	54	**<.0001**
	Activity	498.83 ± 24.76	402.28 ± 27.48	4.339	54	**<.0001**
	Upright posture	437.76 ± 19.15	18.07 ± 7.42	19.640	54	**<.0001**
	Normal posture	280.67 ± 19.17	687.53 ± 13.64	−17.125	54	**<.0001**
All (*N* = 129)	Surface breathing	411.02 ± 15.14	8.13 ± 3.7	25.936	128	**<.0001**
	Activity	435.63 ± 16.64	354.4 ± 19.54	4.472	128	**<.0001**
	Upright posture	432.13 ± 15.94	43.27 ± 9.1	22.214	128	**<.0001**
	Normal posture	276.23 ± 15.11	667.39 ± 10.37	−22.737	128	**<.0001**

The results of statistical comparisons (*t* test for dependent samples) are also shown. *N* number of specimens; *SE* standard error; *df*, degree of freedom. Significant *p* values are in bold.

## 4 DISCUSSION

The results of the two experiments showed that fish‐bearing different homozygous genotypes at the GPI‐1 locus displayed different behaviors when subjected to hypoxic conditions. During the first experiment, the passage of individuals from the hypoxic compartment to the normoxic one was not random, with GPI‐1*85 homozygotes showing a higher propensity to move toward less stressful conditions. In the second test, the two GPI‐1 homozygotes tended to display different behaviors, which were consistent with their nonrandom distribution that emerged in the first test. Among the variables considered in our study, the genotype at GPI‐1 locus appeared to be the only one influencing the behavior of fish, whereas sex, condition index, and sampling group did not significantly affect the observed pattern. These results suggest that similar dynamics could also occur in nature and might contribute to the arising and the maintenance of within‐population genetic divergence. Indeed, a differential ability of genotypes to shy away from a stressful environment could constitute one of the sources of bias in their distribution, with respect to the assumption of random dispersal of genotypes within heterogeneous environments (Edelaar & Bolnick, 2012).

In the first experiment, the majority of individuals moved to the normoxic compartment, despite the small connection size and the relatively short time of 4 hr. This propensity for most individuals to shy away from hypoxia is consistent with the “condition‐dependent dispersal” idea, stating that extreme conditions may induce individuals to move (Edelaar & Bolnick, 2012). In our experiments, this tendency was independent from sex, condition index, and genotype, except the GPI‐1 locus. At the end of the experiment, a significantly higher proportion of the GPI‐1*85 homozygotes had passed into the normoxic compartment, generating the significant differences in allelic and genotypic frequencies observed at this locus in the two sectors of the aquarium. This difference, although significant, was not high in magnitude, but this was expected as: (1) A complete segregation of genotypes is unlikely, even in the presence of matching habitat choice, due to the unavoidable random component in distribution (Edelaar & Bolnick, 2012); (2) this species displays a gregarious behavior (Cavraro et al., 2013); thus, the passage of some individuals (i.e., the GPI‐1*85 homozygotes) might have induced other individuals (i.e., the other two GPI‐1 genotypes) to make the same “decision,” thus smoothing the difference in allelic frequencies among the two sectors.

In the second test, the increased activity of fish in hypoxia represents the expected response to stressful conditions, as demonstrated for other fish species and could also reflect the tendency to escape or increase exploration in search for different conditions (Petersen & Petersen, 1990; Verheyen et al., 1994; Killen et al., 2012). Although fish in normoxia tended to not be active when assuming an upright posture, as demonstrated by the correlations among these parameters, their activity in hypoxia seemed to be a primary reaction performed independently from posture and the correlated surface breathing. This reaction can explain how, in the first test, most individuals left the hypoxic compartment during a limited time span, as increased activity could have also increased the probability of finding the way to the normoxic side of the aquarium.

During the second experiment, the individuals in hypoxia spent most of the time at the surface breathing and in an upright posture, which is a typical response of fish and other aquatic organisms upon hypoxic stress (Petersen & Petersen, 1990; Wannamaler & Rice, 2000; Wu, 2002). However, the GPI‐1 genotypes allocated different times to this behavior, as the GPI‐1*100 homozygotes breathed at the surface for significantly more time than GPI‐1*85 homozygotes. This evidence is consistent with the lower number of GPI‐1*100 homozygotes found in the normoxic compartment at the end of the first experiment: The greater propensity showed by the GPI‐1*100 homozygotes in breathing near the surface has likely reduced their opportunity to migrate toward the normoxic compartment with respect to the GPI‐1*85 homozygotes, given that the hole between the two compartments was about 20 cm below the surface.

One of the specific aims of this survey was to verify whether habitat choice may be one of the factors promoting the genetic heterogeneity recorded at the same allozyme loci in our previous field surveys. Indeed, to our knowledge, there are no experimental studies in vertebrates, which showed a relationship between individual allozymic genotypes and differential behavioral responses related to habitat choice and consequent genetic divergence. Our results refer to a single allozyme marker and can thus provide just an indication on the dynamics involved in generating a pattern of divergence through habitat choice in this species. This study however clearly suggests that it is worth to deepen this topic using a finer approach and more appropriate markers. Indeed, genomics studies showed that the variability of behavioral traits in human and animals is often linked to the variation at single‐nucleotide polymorphism level of multiple genes (Hunt et al., 2007; TAG Consortium, 2010) and that stress can alter brain proteome profile in zebrafish, besides its behavior (Chakravarty et al., 2013). Nevertheless, our study indicates that the arising genetic divergence from a nonrandom dispersal of genotypes among contiguous microhabitats is possible, as suggested by previous allozyme field studies (Byers, 1980, 1983; Borowsky, 1990; Szarowska et al., 1998), and could be mediated by individual differences with a genetic basis in specific behavioral responses, as hypothesized in the context of dispersal and spatial dynamics (Canestrelli et al., 2016).

Field studies already suggested that the active displacement of individuals under stressful conditions, as those observed in coastal lagoons and salterns, may explain part of the genetic divergence over microgeographic scales (Angeletti et al., 2017; González‐Wangüemert & Vergara‐Chen, 2014). Our experimental data support this hypothesis for *A. fasciatus*. Indeed, in the natural context of the Tarquinia saltworks, an increase in activity of individuals in hypoxia could have promoted the hypothesized displacement of individuals away from hypoxic conditions (see Angeletti et al., 2017). Moreover, the different breathing behavior of the two GPI‐1 homozygotes under hypoxia could also explain the divergence revealed by this locus in the field (Angeletti et al., 2009), as in the Tarquinia saltworks, the ponds are frequently connected by partially raised sluice valves, so that the flow of water and fish can occur only at several centimeters of depth.

In conclusion, these data call for a reconsideration of the role of habitat choice in the micro‐ and macro‐ evolutionary dynamics, including theoretical models. With respect to our results, whether there is a direct connection between the physiological role of GPI‐1 in sensitivity to oxygen, the functionality of its genotypes and the behavior displayed by the genotypes themselves is not known, as allozyme markers rarely provide direct indications on the metabolic pathways involved, and the observed pattern may likely involve a linked locus. On the other hand, glucose phosphate isomerase, in addition to have an essential role in carbohydrate metabolism, is a neurotrophic factor for spinal and sensory neurons (Gurney, Heinrich, Lee, & Yin, 1986; Kugler et al., 1998); thus, a direct connection between GPI locus genotypes and the behavior of fish in hypoxia cannot be excluded.

## CONFLICT OF INTEREST

None declared.

## AUTHOR CONTRIBUTIONS

D. Angeletti, C. Sebbio, C. Carere, and A. Carlini designed the study. C. Sebbio and C. Strinati collected the data. D. Angeletti, C. Sebbio, and C. Carere analyzed and interpreted the data with substantial intellectual contributions of G. Nascetti and R. Cimmaruta. D. Angeletti, C. Sebbio, and C. Carere prepared the manuscript, which was critically revised by all the other authors. All authors approved the final manuscript.
