# Supplementary material for: The role of habitat choice in micro‐evolutionary dynamics: An experimental study on the Mediterranean killifish Aphanius fasciatus (Cyprinodontidae)
Source: Ecol Evol. 2017 Nov 1;7(24):10536–45. doi: 10.1002/ece3.3540 (PMC5743487; doi:10.1002/ece3.3540)
Supplement: Supplementary file 1 [file ECE3-7-10536-s001.docx]

Table S1. Principal component matrix across the three morphometric variables for the extraction of the condition index for fish.

| Morphometric variable | PC 1 (70.70%) | PC 2 (25.42%) | PC 3 (3.86%) |
| --- | --- | --- | --- |
| Length | 0.938 | -0.270 | 0.239 |
| Height | 0.609 | -0.247 | -0.242 |
| Weight | 0.933 | 0.793 | 0.006 |
